# Physical Activity Patterns in University Students: Do They Follow the Public Health Guidelines?

**DOI:** 10.1371/journal.pone.0152516

**Published:** 2016-03-29

**Authors:** Filipe Manuel Clemente, Pantelis Theodoros Nikolaidis, Fernando Manuel Lourenço Martins, Rui Sousa Mendes

**Affiliations:** 1 Instituto Politécnico de Viana do Castelo, Escola Superior de Desporto e Lazer, Melgaço, Portugal; 2 Instituto de Telecomunicações, Delegação da Covilhã, Covilhã, Portugal; 3 Department of Physical and Cultural Education, Hellenic Army Academy, Athens, Greece; 4 Polytechnic Institute of Coimbra, Coimbra College of Education, RoboCorp, ASSERT, Coimbra, Portugal; Cinvestav-Merida, MEXICO

## Abstract

Physical activity is associated with health. The aim of this study was (a) to access if Portuguese university students meet the public health recommendations for physical activity and (b) the effect of gender and day of the week on daily PA levels of university students. This observational cross-sectional study involved 126 (73 women) healthy Portuguese university students aged 18–23 years old. Participants wore the ActiGraph wGT3X-BT accelerometer for seven consecutive days. Number of steps, time spent sedentary and in light, moderate and vigorous physical activity were recorded. The two-way MANOVA revealed that gender (*p-value* = 0.001; *η*^2^ = 0.038; *minimum effect*) and day of the week (*p-value* = 0.001; *η*^2^ = 0.174; *minimum effect*) had significant main effects on the physical activity variables. It was shown that during weekdays, male students walked more steps (65.14%), spent less time sedentary (6.77%) and in light activities (3.11%) and spent more time in moderate (136.67%) and vigorous activity (171.29%) in comparison with weekend days (p < 0.05). The descriptive analysis revealed that female students walked more steps (51.18%) and spent more time in moderate (125.70%) and vigorous (124.16%) activities during weekdays than in weekend days (p < 0.05). Women students did not achieve the recommended 10,000 steps/day on average during weekdays and weekend days. Only male students achieved this recommendation during weekdays. In summary, this study showed a high incidence of sedentary time in university students, mainly on weekend days. New strategies must be adopted to promote physical activity in this population, focusing on the change of sedentary behaviour.

## Background

Due to the globally recognised role of physical activity (PA) for the promotion of health and well-being important international organizations and societies have developed guidelines for optimal PA levels [1,2]. According to the World Health Organization, the recommendations about adults aged 18–64 years old were that they should do at least 150 min of moderate or 75 min vigorous intensity aerobic PA weekly and aerobic PA should be performed in bouts of at least 10 min duration [2]. Recent studies have highlighted that university students do not meet current recommendations for PA levels [3–5]. It has been observed that, compared to children and adolescents, university students were less active [3].

Although there has been a lack of comparative studies between student and non-student groups with regards to PA and lifestyle habits, students have attracted special scientific interest because they consist a group with unique characteristics during a crucial period of life, immediately after adolescence. Recent studies highlighted that college students present considerable gains in body mass [6,7]. A specific characteristic of university students is that they undergo emotional, physiological and environmental changes influencing their consumer habits and lifestyle aspects such as PA [8]. A research on 23 countries showed that 40% were physically inactive with scores ranging from 21.9% in Kyrgyzstan to 80.6% in Pakistan [4]. Accordingly, 60% [3] to 72.6% of Spanish universtiy students did not meet the guidelines [5]. In addition to the low number of those who meet the PA guidelines, the abovementioned studies indicated a large variation of observations from study to study. Nevertheless, these studies have relied mostly on self-report measures of PA (i.e. questionnaire) [3–5] and less information is available by research using an objective measure such as accelerometer.

Accelerometer has been proved to be a valid objective measure of PA in university students [9]. On the other hand, compared to accelerometer, self-report measures have been shown to overestimated moderate and vigorous PA [10]. Moreover, accelerometers can provide detailed information about time spent on different intensities. Nevertheless, only a few data exist on patterns of PA assessed by accelerometer in university students. For instance, a study using accelerometer for seven consecutive days on USA university students showed that they were more active during weekdays than weekend and that males were more active than females in total and moderate PA scores, but there was not any gender-related difference with regards to time spent to moderate or vigorous PA sessions of at least 10 min [11], However, these findings have not been confirmed in other samples. Research on adolescents has suggested that the variation of PA by days of week was attributed to different correlates between weekdays and weekend [12,13] highlighting the importance of family and community support.

Although the abovementioned studies have enhanced our understanding of PA patterns, there is a lack of data concerning PA patterns of different intensities during weekdays and weekend in female and male university students. Another drawback of these studies was that they relied mostly on self-report measures of PA indicating the need for studies using objective measures, such as accelerometer. For instance, it has been shown that self-report questionnaires might not provide accurate information about the type, intensity and amount of PA in university students [14]. Such information about these patterns might be of practical importance for both sports scientists and health-related practitioners. For instance, knowledge about PA patterns in female and male university students would help to develop gender-tailored PA interventions. Moreover, being aware of differences in PA between weekdays and weekend would contribute to develop PA programs according to its daily variation. Therefore, the aim of the present study was to examine (a) whether Portuguese university students meet the public health recommendations for PA and (b) the effect of gender and day of the week on daily PA levels of university students. The focus of this study was quantitative rather than qualitative aspects of PA, i.e. the type of PA performed (occupational, leisure-time, household chores or transport) was not considered.

## Methods

### Study Design and Population

**Fig 1:**
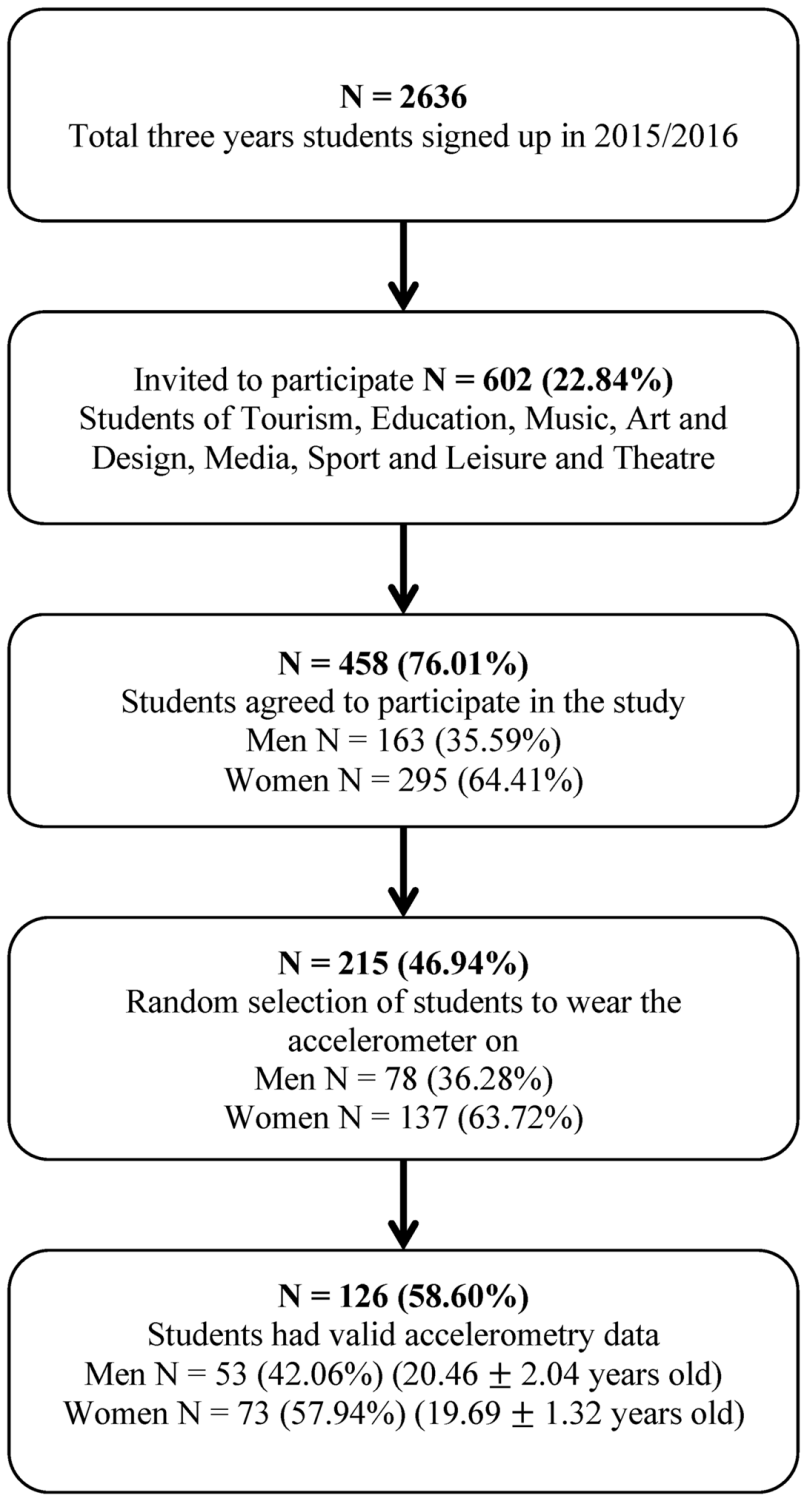
Flowchart showing the progress of students during the study.

The measurements of this study were conducted in 2015–2016 in the city campus (IPC-ESEC, Portugal). From a total of 861 students in three years, 602 students were invited to participate. They were all from six different degrees (Tourism, Education, Music, Art and Design, Media, Sport and Leisure and Theatre) and only four of five degrees were randomly invited to participate (Fig 1). Only the Sport and Leisure course was related to the PA. From the 602 invited students, 458 (76.01%) agreed to participate.

The investigation attended first, second and third year courses to explain the aims of the study and invite participants; those interested signed the informed consent. Students received an academic reward for having participated in the study.

The university population included students older than 17 years old. Most of them lived in rented flats (63%) or in the campus hostels (19%) close to the campus, so they are not far away from the lecturing places. From the 126 participants, 33 (26.19%) were amateur athletes.

The study protocol was approved by the Research Ethics Committee of Polytechnic Institute of Coimbra, code: 01.09/2015 and all subjects signed informed consent forms prior to their participation in the study. The study followed the ethical recommendations for the study in humans as suggested by the Declaration of Helsinki.

### Anthropometry

The measurements were made in the University by trained researchers to minimize the inter-observer variability. Body mass and height were measured following standardized recommendations with an electronic scale (Tanita SC 330 S; precision = 100 g, range = 0–270 kg), and a stadiometer (Type SAGE, precision 0.1 cm, range = 0–230 cm). Tanita body composition analyser measures body composition using a constant current source with a high frequency current (50kHz). The 8 electrodes are positioned so that electric current is supplied from the electrodes on the tips of the toes of both feet, and voltage is measured on the heel of both feet. Tanita has developed the technology for measuring the visceral adipose tissue accumulation risk through bioelectrical impedance analysis in comparison with image analysis applied to magnetic resonance imaging (MRI). Despite the bioeletrical impedance analysis not be the most appropriate choice for body composition measurement, appropriate algorithms for population can accurately measure body fat [15]. This device is recurrently revised in relation to the reference standards DXA and has been validated against other weighing methods [16,17]. Study of physical activity patterns have been used similar body composition analyser to determine %Fat mass, Fat mass, Muscle Mass and Bone mass [17–19]. Body mass index (BMI) was calculated as body mass (kg) divided by height^2^ (m).

### Assessment of physical activity

Participants wore an accelerometer (model wGT3X-BT, ActiGraph, Shalimar, FL, USA) for seven consecutive days. This equipment has been shown to be an objective valid measure of body movement in a study on university students that had examined it in nine 5-min semi-structured activities (lying, sitting, reading, seated video gaming, video watching, seated conversation, standing, stationary biking and treadmill walking) [9]. We used a 20-min consecutive zero count to define non-wearing time, following the design of similar studies [20,21]. Wearing time was calculated by subtracting non-wearing time from 24 hours [22]. To be considered valid data the participants should use the accelerometer in 90% of the time, thus ensuring that the device it assessed all the activity and inactivity of students. The device was worn on the right hip of the participants, attached with an elastic band. Because the accelerometers were not water-resistant, the subjects were asked to remove them before showering or entering a pool. The interval of recorded time (epoch) was set at 60 seconds, shown as valid for measuring PA in adults. Data were collected throughout the school year (October to February). The accelerometer measured the activity profile by using a Freedson Adult algorithm [23] and the wear time information by using Troiano’s algorithm [22]. Accelerometer data were processed (ActiLife 6.0.) to provide values for total daily and hourly counts per minute (cpm) sedentary time (minutes and %), light PA (minutes and %), moderate PA (minutes and %), vigorous PA (minutes and %), moderate-to-vigorous PA and steps for weekdays and weekend. The intensity of weekly PA was assessed as average cpm. The cut-off values used to define the intensity of PA and, therefore, quantify the mean time in each intensity were the following sedentary time = < 100 cpm, light PA = 100–1951 cpm, moderate PA = 1952–5724 cpm and vigorous PA = >5725. These cut-off values followed the study that tested university students in Spain by using accelerometers [18].

### Statistical Procedures

In this study the gender (male and female) and the moment of the week (weekdays and weekend) were defined as factors. The anthropometric variables (Weight, Height, BMI, %Fat mass) and PA variables (sedentary, light, moderate, vigorous, in minutes and number of steps) were defined as dependent variables. S1 Dataset provides the data used in this study. The two-way MANOVA was used after validating normality and homogeneity assumptions. When the MANOVA detected significant statistical differences between the two factors, we proceeded to the two-way ANOVA for each dependent variable, followed by Tukey’s HSD post-hoc test [24]. Ultimately, the statistical procedures used were one-way ANOVA and Tukey HSD post-hoc per factor. Effect size (ES) was presented as *η*^2^ and interpreted using the follow criteria: no effect (*η*^2^ < 0.04), minimum effect (0.04 < *η*^2^ < 0.25), moderate effect (0.25 < *η*^2^ < 0.64) and strong effect (*η*^2^ > 0.64) [25]. All data sets were tested for each statistical technique and corresponding assumptions and performed using SPSS software (version 23.0, Chicago, Illinois, USA). Statistical significance was set at 5%.

## Results

Anthropometric characteristics are displayed in Table 1. There were statistical differences between men and women in height (p-value = 0.001; *η*^2^ = 0.158), weight (p-value = 0.001; *η*^2^ = 0.190), %fat mass (p-value = 0.001; *η*^2^ = 0.551) and muscle mass (p-value = 0.001; *η*^2^ = 0.685). Descriptive statistics revealed that male participants are taller (8.7%), heavier (16.6%) and have more muscle mass (39.6%) than female participants. By the other hand, female participants have a statistically greater percentage of fat mass (58.4%) than male participants. No statistical differences in BMI were found between male and female participants.

**Table 1:** Descriptive anthropometric characteristics of the studied participants.

	Gender	M(SD)	p-value	Effect Size (ES)	CI (95%)
Height (cm)	Male	176.66(7.20)^b^	0.001	0.158 *Minimal effect*	[175.30–178.30]
	Female	162.52(20.26)^a^			[161.12–163.92]
Weight (kg)	Male	71.52(10.60)^b^	0.001	0.190 *Minimal effect*	[70.46–72.58]
	Female	61.34(10.24)^a^			[60.44–62.25]
%Fat mass	Male	10.30(4.56)^b^	0.001	0.551 *Moderate effect*	[9.65–10.96]
	Female	24.73(7.54)^a^			[24.16–25.29]
Muscle Mass (kg)	Male	60.67(7.35)^b^	0.001	0.685 *Strong effect*	[60.09–61.26]
	Female	43.45(4.27)^a^			[42.95–43.95]
BMI (kg.m^-2^)	Male	22.57(2.66)	0.776	0.001 *No effect*	[22.25–22.90]
	Female	22.64(3.56)			[22.36–22.92]

Statistically different from male^a^;

and female^b^ for a p-value < 0.05.

The two-way MANOVA revealed that the gender (*p-value* = 0.001; *η*^2^ = 0.038; *minimum effect*) and moment of the week (*p-value* = 0.001; *η*^2^ = 0.174; *minimum effect*) had significant main effects on the PA variables. There was significant interaction (Pillai’s Trace = 0.020; *p* = 0.008; $\eta_{p}^{2}$ = 0.020; *no effect*) between the gender and the moment of the week. As previously indicated in the statistical procedures, a two-way ANOVA was conducted for each dependent variable after the confirmation of the interaction (O’Donoghue, 2012, p. 243).

Interaction was found between factors for steps (*p-value* = 0.031; *η*^2^ = 0.005; *no effect*), time of sedentary activity (*p-value* = 0.028; *η*^2^ = 0.006; *no effect*), time of light activity (*p-value* = 0.030; *η*^2^ = 0.005; *no effect*) and time of vigorous activity (*p-value* = 0.001; *η*^2^ = 0.013; *no effect*). No statistically interactions were found in the time of moderate activity (*p-value* = 0.060; *η*^2^ = 0.004; *minimum effect*).

Table 2 shows the descriptive statistics about the PA patterns of students during one week using accelerometer.

**Table 2:** Descriptive physical activity characteristics of the studied participants and the comparisons between genders and moment of the week.

	Men (n = 53)	Women (n = 73)	Overall (n = 126)	p-value	ES
Weekday PA					
*Steps*	11272.58(8269.53)*	9096.73(3955.35)*	10011.97(6237.90)*	0.001	0.030 *No effect*
CI (95%)	[10382.51–12162.66]	[8704.07–9489.39]	[9569.40–10454.54]		
ES	0.070—*minimum effect*	0.088—*minimum effect*	0.068—*minimum effect*		
*Sedentary time (min/day)*	725.93(177.97)*	769.29(150.10)	751.05(163.68)	0.001	0.017 *No effect*
CI (95%)	[703.15–748.72]	[753.19–785.38]	[737.64–764.47]		
ES	0.016—*no effect*	0.001—*no effect*	0.003—*no effect*		
*Light PA (min/day)*	301.30(96.83)	279.65(76.18)*	288.76(86.07)	0.002	0.015 *No effect*
CI (95%)	[288.36–314.25]	[271.28–288.02]	[281.45–296.07]		
ES	0.001—*no effect*	0.014—*no effect*	0.002—*no effect*		
*Moderate PA (min/day)*	64.99(42.89)*	51.33(33.82)*	57.08(38.46)*	0.001	0.031 *No effect*
CI (95%)	[60.23–69.75]	[48.12–54.54]	[54.32–59.84]		
ES	0.157—*minimum effect*	0.138—*minimum effect*	0.142 *minimum effect*		
*Vigorous PA (min/day)*	5.67(8.80)*	1.65(3.39)	3.34(6.56)*	0.001	0.091 *Minimum effect*
CI (95%)	[4.72–6.62]	[1.28–2.03]	[2.87–3.81]		
ES	0.041—*minimum effect*	0.005—*no effect*	0.019—*no effect*		
Weekend Days PA					
*Steps*	6826.07(4343.33)*	6474.48(3450.74)*	6622.37(3847.17)*	0.475	0.002 *No effect*
CI (95%)	[5418.73–8233.40]	[5853.63–7095.33]	[5922.60–7322.14]		
ES	0.070—*minimum effect*	0.088—*minimum effect*	0.068—*minimum effect*		
*Sedentary time (min/day)*	778.72(72)*	765.18(171.59)	770.87(189.88)	0.577	0.001 *No effect*
CI (95%)	[742.69–814.75]	[739.73–790.63]	[749.66–792.08]		
ES	0.016—*no effect*	0.001—*no effect*	0.003—*no effect*		
*Light PA (min/day)*	292.19(129.52)	301.05(93.32)*	297.33(109.85)	0.528	0.002 *No effect*
CI (95%)	[271.72–312.65]	[287.81–314.30]	[285.77–308.88]		
ES	0.001—*no effect*	0.014—*no effect*	0.002—*no effect*		
*Moderate PA (min/day)*	27.46(28.81)*	23.71(23.33)*	25.29(25.79)*	0.254	0.005 *No effect*
CI (95%)	[19.94–34.99]	[18.63–28.78]	[20.92–29.65]		
ES	0.157—*minimum effect*	0.138—*minimum effect*	0.142—*minimum effect*		
*Vigorous PA (min/day)*	2.09(4.70)*	1.05(4.27)	1.49(4.48)*	0.069	0.013 *No effect*
CI (95%)	[0.59–3.59]	[0.46–1.65]	[0.75–2.24]		
ES	0.041—*minimum effect*	0.005—*no effect*	0.019—*no effect*		

*Statistically differences between weekdays and weekend days for a p-value < 0.05.

The analysis of variance between moment of the week revealed that in male students there were statistical differences in number of steps (p-value = 0.001; *η*^2^ = 0.070), sedentary time (p-value = 0.001; *η*^2^ = 0.016), moderate activity (p-value = 0.001; *η*^2^ = 0.157) and vigorous activity (p-value = 0.001; *η*^2^ = 0.041). In this case, it was possible to also verify that during weekdays male students walked more steps (65.14%), spent less time in sedentary (6.77%) and light activities (3.11%) and spent more time in moderate (136.67%) and vigorous activity (171.29%) in comparison with weekend days. In the case of female students, it were found statistical differences between moment of the week in number of steps (p-value = 0.001; *η*^2^ = 0.068), moderate activity (p-value = 0.001; *η*^2^ = 0.142) and vigorous activity (p-value = 0.001; *η*^2^ = 0.019). The descriptive analysis revealed that female students walked statistically more steps (51.18%) and spent more time in moderate (125.70%) and vigorous (124.16%) activities during weekdays than in weekend days.

The comparison between genders activities during weekdays revealed statistical differences in number of steps (p-value = 0.001; *η*^2^ = 0.030), sedentary time (p-value = 0.001; *η*^2^ = 0.017), light activity (p-value = 0.002; *η*^2^ = 0.015), moderate activity (p-value = 0.001; *η*^2^ = 0.031) and vigorous activity (p-value = 0.001; *η*^2^ = 0.091). It was also possible to verify that male students walked statistically more steps (23.92%) and spent more time in light (7.74%), moderate (26.61%) and vigorous (243.64%) activities than female students. By the other hand, female students spent statistically more time in sedentary time (5.64%). No statistical differences were found in the case of PA patterns made by both genders during weekend days.

## Discussion

There is a lack of studies analysing the PA patterns in university students, particularly in Portugal. Studying a specific population like university students is extremely interesting considering the abrupt change that this population is subject to especially with regards to behaviour and lifestyle after passing from the well-controlled environment of high school to independent habits on college [18,26]. Our data reveal that the amount of PA that Portuguese university students perform complies the recommendation of moderate-to-vigorous PA for most of the week (5 days) [2,27].

Previous studies conducted in Portuguese population revealed that 70% of participants with age between 18–29 reached the recommendation of 30 min/day of PA, when counting every minute of moderate or greater intensity [28]. In the particular case of university students, the values revealed inactivity patterns in 41% of the men and 65% of the women students [29]. In our study it was possible to find that in women population it was achieved an average of 51.33 minutes of moderate activity and 1.65 minutes of vigorous activity per day during weekdays. In men students the values were higher, particularly achieving 64.99 minutes of moderate activity and 5.67 minutes of vigorous activity per day during the weekdays. Despite of these values, it was observed a decrease of activity profile from weekdays to weekend days in both genders. In male students, it was observed a statistically greater activity in moderate-to-vigorous intensity during weekdays between 136% and 171% comparing to weekend days. Similar results were found for female students. Both results follows the evidences revealed in a study conducted in Spanish university students [18].

The comparison made between genders revealed that male students walked statistically more steps (23.92%) and spent more time in light (7.74%), moderate (26.61%) and vigorous (243.64%) activities than female students. Previous studies revealed that this evidence are usual in the majority of the countries [28,30]. Probably, cultural practices and habits from children can be associated with the prevalence of activity per gender [31]. Despite of these results, in our study the statistical differences were only found during weekdays. On weekend days there were no statistical differences between genders, thus suggesting that they may have similar activity habits during such moment of the week.

Our study did not considered the 10 minutes bouts recommendations for moderate-to-vigorous activities. In fact, this guideline can be very important criteria to analyse the PA patterns of university students. In a study conducted in Portuguese population it was showed that when blocks of 10 minutes of continuous activity of moderate or vigorous intensity were considered, the prevalence of the active population dramatically decreased, varying from ~70% of people aged 18–29 to ~5% accumulating at least 30 minutes of PA per day [28]. In fact, the prevalence of more continuous activities seems to be greater in older population (40 to 64 years old) and the more intermittent activities (but maybe more intense) seems to be associated with younger adults [18]. In fact, some fitness levels can be improved without a continuous workout, such as cardiorespiratory fitness and health variables [32].

The context of physical activity it was not analysed in this study. Nevertheless, this topic should be carefully considered in further researches. A study that determined the physical activity contexts in university students revealed that male and female students preferred exercising with others outside of a structured class [33]. Such evidence was also identified in similar studies [34,35]. Moreover, the environmental factors can also influence physical activity patterns. An interesting study found that students living in fraternity (groups) tended to be more actives and participate in more sports activities, thus increasing the time spent in moderate-to-vigorous activity [36]. The use of social support by using online services (online social networking) have been also revealing interesting results to increase physical activity patterns of university students [37]. For these reasons, the benefits of sharing activities among colleagues should be seriously considered by government policies and health services to promote physical activity in this specific population.

Besides the previous described experimental limitation, it is also possible to speculate that our study may be affected by the Hawthorne effect based on the fact that participants only were wearing the device for one-week. Another limitation that can be affected this study was the uncontrolled factors associated with economic levels or weight status. The third limitation may also be the percentage of amateur athletes included in this study. The high percentage may have influenced the outcomes of this study. Despite of such limitations, this study revealed some similarities with some studies conducted in Portuguese population, thus suggesting that the evidences can be associated with the specific PA patterns registered in this country. In comparison with other countries, it was found greater participation of students in moderate-to-vigorous activities, even comparing with the neighbour country, Spain. Future studies must consider the time of activities made by students and the bouts performed by the students during their activities. Analyse the variance between populations with or without regular practice of exercise it will be also important.

The findings of the present study have important practical applications for exercise specialists and other professionals who are involved in the development of large-scale PA programs in universities. The focus of such programs should be to increase general PA levels especially during weekends. Moreover, an emphasis should be given to female students. Results obtained in Portugal (our study) and Spain [18] should be a serious warning to the health services of both countries. Current facilities and equipment may not be attractive for university students. Moreover, the facilities do not ensure the enough motivation to university students [38]. Governmental policies must attract students for physical activity. Each institution may offer low-cost or free programs of physical activity for university students. Moreover, governments for this specific population must study policies of reward. Regular use of low-cost bicycles provided by institutions can be interesting to increase physical activity levels during house-to-campus travels. Walking routes for the cities can be also interesting, mainly combining the walking with culture. Short periods without classes in the morning and afternoon (~30 minutes) may be also used to promote regular events such as master classes or similar activities that can motivate students to share the practice with their colleagues. Finally, online communities can be also interesting to promote the support or challenges during activities and to share experiences among colleagues.

## Conclusions

In this study it was possible to verify that in average both young men and women Portuguese university students complies the PA recommendations during weekdays with more than 10,000 steps and 30-minutes of moderate-to-vigorous PA per day. Different results were found during weekend days, not achieving the PA recommendations. It was also possible to observe statistical differences in PA patterns between male and female university students during weekdays. No statistical differences between genders were found during weekend days. Future studies must adopt an analysis comparing bouts of activity, trying to verify if the 10-minutes of continuous activity is accomplished or to analyse if there are a tendency to intermittent activities during the day. Implications for government policies in this specific population must be considered, mainly promoting group activities and reward policies.

## Supporting Information

S1 Dataset Dataset used in for statistical procedures. First column: Day of the week; Second column: Weekdays or weekends; Third column: Gender; Fourth column: Number of steps; Fifth column: Minutes in sedentary mode; Sixth column: Minutes in light activity; Seventh column: Minutes in moderate activity; Eighth column: Minutes in vigorous activity.
(XLSX)
